# Contribution of a GATA4-Expressing Hematopoietic Progenitor Lineage to the Adult Mouse Endothelium

**DOI:** 10.3390/cells9051257

**Published:** 2020-05-19

**Authors:** Rita Carmona, Sandra Díaz del Moral, Silvia Barrena, Ramón Muñoz-Chápuli

**Affiliations:** 1 Department of Animal Biology, Faculty of Science, University of Málaga, 29071 Málaga, Spain; rita@uma.es (R.C.); sandra.ddm@outlook.com (S.D.d.M.); silviabarrenagarcia@gmail.com (S.B.); 2 Andalusian Center for Nanomedicine and Biotechnology (BIONAND), 29590 Málaga, Spain; 3 Institute of Biomedical Research of Málaga (IBIMA), 29010 Málaga, Spain

**Keywords:** circulating endothelial progenitors, coronary vessels, vascular growth, endothelium

## Abstract

Different sources have been claimed for the embryonic origin of the coronary endothelium. Recently, the potential of circulating cells as progenitors of the cardiac endothelium has also been suggested. In a previous study we have shown that circulating progenitors are recruited by the embryonic endocardium and incorporated into the coronary vessels. These progenitors derive from a mesodermal lineage characterized by the expression of *Gata4* under control of the enhancer G2. Herein, we aim to trace this specific lineage throughout postnatal stages. We have found that more than 50% of the adult cardiac endothelium derives from the G2-GATA4 lineage. This percentage increases from embryos to adults probably due to differential proliferation and postnatal recruitment of circulating endothelial progenitors. In fact, injection of fetal liver or placental cells in the blood stream of neonates leads to incorporation of G2-GATA4 lineage cells to the coronary endothelium. On the other hand, labeling of the hematopoietic lineage by the stage E7.5 also resulted in positive coronary endothelial cells from both, embryos and adults. Our results suggest that early hematopoietic progenitors recruited by the embryonic ventricular endocardium can become the predominant source of definitive endothelium during the vascularization of the heart.

## 1. Introduction

Endothelial cells primarily differentiate from angioblasts in a process called vasculogenesis. This process accounts for the formation of the earliest vessels in the embryo. Later, vascular growth is mainly achieved by angiogenesis, i.e., sprouting of pre-existing vessels. Vascular growth can also occur during development by recruiting angioblasts resident in the tissues [1] or circulating endothelial progenitor cells (EPC) [2]. The existence of circulating EPC has been well characterized in adults [3]. EPC can be mobilized from the bone marrow in response to injury and they can incorporate into the endothelium of damaged areas. However, there are few data concerning the existence of embryonic circulating endothelial progenitors and their putative participation in the vascularization of the mammalian embryo. For example, it is known that the murine fetal liver contains hematovascular progenitors with endothelial engraftment potential when transferred to the blood stream of newborns or adult conditioned recipients [4]. We have reported elsewhere that 20% of all the adult hematopoietic system in mice derives from progenitors where the GATA4 gene expression is driven by the specific mesodermal enhancer G2. Most of these progenitors arise in the placenta. Transplantation of fetal G2-GATA4 lineage cells into busulfan-treated newborns gives rise to endothelial cells at least in the liver [5]. More recently, it has been shown that an embryonic population of erythro-myeloid progenitors colonizes the embryo and contributes endothelial cells to the endothelium in multiple organs where they persist until adulthood [2]. This study estimated that 15% of the cardiac endothelium arises from the erythro-myeloid progenitors described by them. On the other hand, the existence of circulating endothelial progenitors in avian embryos has been demonstrated [6,7].

Thus, circulating progenitors constitute a potential source of endothelial cells for vascular growth during development, but their relevance for the establishment of the coronary circulation is still poorly known. Evidence for an origin of the coronary endothelium from different sources has been proposed in recent years, including subpopulations of proepicardial cells [8,9], ventricular endocardium [10], sinus venosus endocardium [11,12,13] and regionally distributed contribution of ventricular endocardium (ventral wall) and sinus venosus endocardium (dorsal wall) with a minor contribution of the epicardium [14]. The mosaic nature of the coronary endothelium is probably the only existing consensus in this regard, as it has been extensively discussed in recent reviews [15,16]. It is particularly debated if mesenchymal cells derived from the epicardium can contribute to the coronary endothelium. We have recently studied the developmental fate of four cell lineages related with the embryonic epicardium using the Cre-Lox technology [17]. In that report we showed that two epicardial-related cell lineages, revealed by the GATA5Cre and the cTnTCre drivers, accounted for about 4% of the cardiac endothelium along the gestation, probably representing the actual epicardial contribution to the coronary endothelium in embryos. The WT1 cell lineage is also related with the embryonic epicardium, where this gene is highly expressed, and its endothelial contribution reaches 7% at the end of gestation, probably due to de novo expression in non-epicardial-derived cells (EPDC). The G2-GATA4^Cre^; R26R^eYFP^ model shows recombination in most of the epicardium and the EPDC. Thus, we expected a similar percentage of cardiac endothelial cells than the obtained with the other three models, but we found that this percentage increased sharply by midgestation from 5% to 20% of all the cardiac endothelium. Most surprisingly, many G2-GATA4 endothelial cells appeared frequently associated with the endocardium, far away from the epicardium. Given our observations on the presence of the G2-GATA4 lineage in the hematopoietic cells, we concluded that this increase was due to recruitment of G2-GATA4+ circulating endothelial progenitors into the endocardium. We also found evidence that these G2-GATA4+ endothelial progenitors proliferate faster than other endothelial cells, thus explaining the sharp increase of this population during gestation.

New observations performed on neonates and adults have shown that this increase in the participation of G2-GATA4 lineage endothelial cells in the cardiac vascularization continues postnatally giving rise to more than 50% of the cardiac endothelium in adults. We have also detected the presence of this lineage in the endothelium of other organs. In fact, this lineage appears as predominant in the adult kidneys. The aim of this study is to describe the contribution of the G2-GATA4 cell lineage to the endothelium of different adult organs. Our findings stress the importance of embryonic circulating endothelial progenitors in the definitive vascularization of the heart.

## 2. Materials and Methods

The animals used in our research program were handled in compliance with the institutional and European Union guidelines for animal care and welfare. The procedures used in this study were approved by the Committee on Ethics of Animal Experiments of the University of Malaga (procedure code 2018-0018). The adult mice used in this study were aged between three and six months.

The Tg(WT1-cre)#Jbeb (WT1^cre^) mouse line has been used in previous studies to trace the WT1-expressing cell lineage or to delete specific genes in WT1-expressing cells [9,18,19,20,21]. For the generation of a G2-GATA4^Cre^ line, a 642-base-pair fragment containing the minimal promoter from the mouse *Mef2c* gene was cloned into a plasmid containing the Cre complementary DNA and the SV40 splice and polyA signal sequence. An 817-bp fragment containing the conserved region, CR2, of the previously identified mouse G2 *Gata4* enhancer was then cloned into the Cre expression vector to generate G2-GATA4^Cre^ transgene [22,23]. Details of the GATA5^Cre^ model have been published [24]. For cardiac troponin T lineage tracing we have used the Tg(Tnnt2-cre)5Blh mouse strain. The TnT promoter drives early expression of Cre recombinase in the cardiomyocyte lineage beginning at E7.5 [25,26,27].

For lineage tracing studies, the murine lines carrying the Cre drivers (except for the GATA5^Cre^ line) were crossed with homozygote Rosa26^EYFP^ (B6.129X1-Gt(ROSA)26Sor^tm1(EYFP)Cos^/J) mice to generate permanent reporter expression in the lineage of Cre-expressing cells. The GATA5^Cre^ mice were crossed with a Gt(ROSA)26Sor^tm1Hjf^ reporter allele for Cre activity that expresses a non-toxic tandem–dimer red fluorescent protein.

Embryos were staged from the time point of vaginal plug observation, which was designated as the stage E0.5. Whole embryos were excised, washed in PBS and dissected to obtain the hearts or fixed in 4% fresh paraformaldehyde solution in PBS for 2–8 h. Fixed embryos were paraffin-embedded or washed in PBS, cryoprotected in sucrose solutions, embedded in OCT and frozen in liquid N_2_-cooled isopentane. Ten μm cryosections were stored at −20 °C until use.

### 2.1. Analytical Flow Cytometry

For flow cytometry analysis, dissected hearts from embryos or neonates were dissociated for 20 min at 37 °C in pre-warmed 0.1% collagenase solution in cytometry buffer (PBS plus 2% fetal bovine serum and 10-mM HEPES) and homogenized by repeated pipetting. Adult hearts were finely minced and treated with Liberase TM (Merck KGaA, Darmstadt, Germany) for seven minutes at 37 °C. After homogenization by repetitive pipetting, the cell suspension was mixed with 4:1 vols of neutralizing buffer (5% sheep serum in DMEM), centrifuged and resuspended in cytometry buffer. In both cases, cell suspensions were washed in cytometry buffer and filtered through a 70-μm nylon mesh. Then, cells were incubated on ice in the dark with the fluorochrome-conjugated antibodies. DAPI staining showed that less than 10% of the cells were damaged by this protocol.

Cells were analyzed in a FACS Verse flow cytometer. Data were analyzed with Kaluza Analysis software 2.1 (Beckman Coulter, Indianapolis IN 46268, USA) and displayed on tables as mean ± standard error of mean. Negative controls (Cre-negative littermates) and isotypic antibodies allowed setting of the gates.

Details of the antibodies used for flow cytometry are provided in Table S1.

### 2.2. Immunofluorescence and Confocal Microscopy

Immunofluorescence was performed using routine protocols. Deparaffinized sections or cryosections were rehydrated in Tris-PBS (TPBS) and blocked for non-specific binding with SBT (16% sheep serum, 1% bovine albumin, 0.1% Triton X-100 in TPBS). When biotinylated secondary antibodies were used, endogenous biotin was blocked with the Avidin-Biotin blocking kit from Vector. Single immunofluorescence was performed incubating the sections with the primary antibody overnight at 4 °C, washing in TPBS and incubating with the corresponding fluorochrome-conjugated secondary antibody. Double immunofluorescence was performed by mixing both primary antibodies (rabbit polyclonal and mouse or rat monoclonal) and incubating overnight at 4 °C. We then used a TRITC-conjugated and a biotin-conjugated secondary antibody, followed by 45 min incubation with Cy5-conjugated streptavidin. Nuclei were counterstained with DAPI (Sigma, D-4592).

Details of the antibodies used for immunofluorescence are provided in Table S1.

### 2.3. Placenta/Fetal Liver Chimaeras

Busulfan (1,4-Butanediol dimethanesulfonate, Merck KGaA, Darmstadt, Germany) is a myeloablative agent used to improve the efficiency of a hematopoietic graft in newborn mice [5,28]. Pregnant females were treated by intraperitoneal (i.p.) injection on days 17 and 18 of gestation with a 15 mg busulfan/Kg dose. Newborns at stage P1 are injected through the facial vein with cells suspended in 50 μL of DPBS + 1% FCS + 1% penicillin/streptomycin (P/S). The cells were obtained from E11.5 G2-GATA4^Cre^; R26R^EYFP^ embryos by mechanical disaggregation of fetal livers or collagenase treatment of placentas (0.12% collagenase in Ca^2+^ and Mg^2+^ free-PBS supplemented with 10% FCS and 1% P/S, 30 min at 37 °C). After washing, the cell suspension was filtered, centrifuged and resuspended in the injection buffer. Two and four newborns were injected with fetal liver and placental cells, respectively.

### 2.4. Labeling of the SCL Lineage

The tamoxifen inducible HSC-SCL^CreERT^; R26R^EYFP^ [29] pregnant mice were used for permanent labeling of the early hematopoietic progenitors. Reporter expression was induced by the stage E7.5 by i.p. injection with tamoxifen (Merck KGaA, Darmstadt, Germany, T5648) dissolved in corn oil (10 mg/mL) at a dose of 0.1 mg/g body weight, together with 0.05 mg/g body weight of progesterone to reduce abortion risk. The embryos were fixed at the stage E14.5 and processed for flow cytometry and confocal microscopy. An adult mouse, four weeks old, induced at the stage E7.5, was also processed for flow cytometry of the heart.

## 3. Results

### 3.1. The G2-GATA4 Cell Lineage Constitutes a Major Part of the Adult Cardiac and Kidney Endothelium

**Figure 1:**
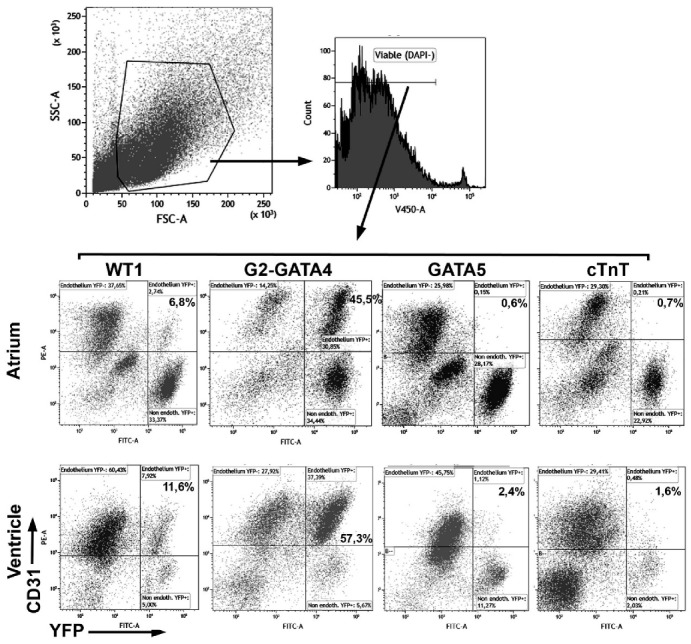
Representative cytograms of the contribution of the four lineages studied to the adult cardiac endothelium. Percentages on the cytograms refer to total viable cells (DAPI-negative) of the endothelial (CD31+) subpopulations derived from each lineage. A major part of the endothelial cells in atrium and ventricle derives from a G2-GATA4 lineage, as detailed in Table 1.

We have compared the endothelial and non-endothelial adult fate of four cell lineages related with the embryonic epicardium. The results are shown in Table 1. The gating strategy and representative cytograms from all the lineages are shown in Figure 1. Endothelial cells were overrepresented in the cell suspensions after the enzymatic treatment.

**Figure 2:**
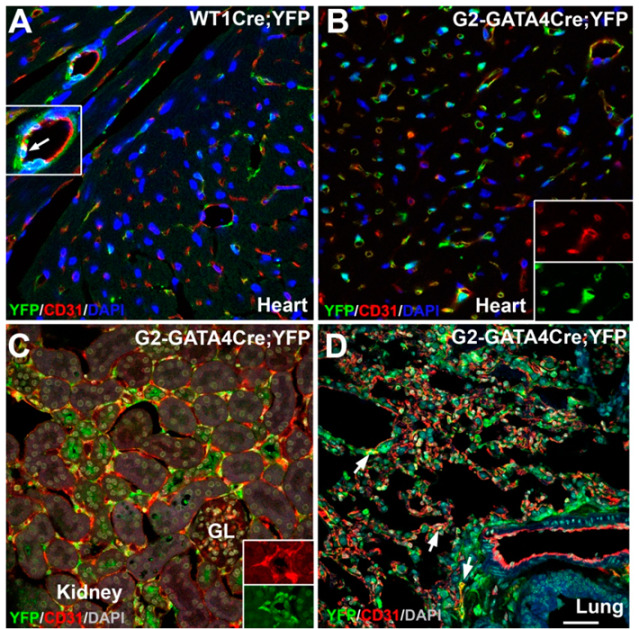
Localization of endothelial cells of the WT1 and G2-GATA4 lineages in adult heart, kidney and lung. In the heart, the WT1 lineage endothelium (**A**) is less abundant than the G2-GATA4lineage endothelium (**B**). Most of the kidney endothelium is also derived from the G2-GATA4 lineage (**C**), but this endothelium is less frequent in the lung (arrows in **D**). Inserts show higher magnification or separate channels to illustrate colocalization of markers. GL: glomerulus. Scale bar for all the figures: 50 μm.

The WT1 cell lineage contributes to 9.9% of the ventricular and 7.9% of the atrial endothelium in adult mice (Table 1 and Figure 2A). The GATA5 and the cTnT lineages only account for 0.5%–1.5% of the cardiac endothelial cells. In contrast, the G2-GATA4 lineage represents 55.2% of the atrial and 53.3% of the ventricular endothelial cells. We compared this contribution of the WT1 and G2-GATA4 lineages to the cardiac endothelium with that from neonatal mice (Table 2). The percentage of ventricular endothelium from the WT1 lineage was similar to that found in adults (10.4% versus 9.9% in the ventricle). The G2-GATA4 lineage contributed less to the neonatal ventricular endothelium than to the adult ventricular endothelium (34.5% versus 53.3%).

We also studied the contribution of the four lineages to the cardiac non-endothelial cells, basically fibroblasts and perivascular cells characterized by the expression of CD90 and CD140a/PDGFRα (Table 1). The G2-GATA4 and the GATA5 lineages were predominant in these populations, with percentages between 40%–65%, while the WT1 and cTnT lineages showed lower frequencies, between 14%–40% in the ventricle. Despite the differences between the lineages (discussed below), these data confirm the significant contribution of the epicardial-derived cells to the stromal component of the adult heart.

We checked the frequency of endothelial cells from the WT1 and G2-GATA4 lineages in other organs obtained from adult mice (Table 2 and Figure 2). Endothelial cells from the WT1 lineage were less frequent in lung and liver than in the heart (4–6% of all the endothelial cells), and they were virtually absent from the kidney endothelium. The G2-GATA4 lineage accounted for a moderate fraction of the lung (15.6%) and liver (9.9%) endothelium, but this percentage was predominant in the kidney (86.7%). We also studied the contribution of the WT1 and G2-GATA4 lineages to the liver, lung and kidney endothelium of neonatal mice. The WT1 lineage increases moderately in the endothelium of lung and liver, but this lineage is virtually lacking in the endothelium of the neonatal kidney. The endothelial contribution of the G2-GATA4 lineage increases mildly in the lung and it does not increase in kidneys (where it already accounts for 92.7% of all endothelial cells in neonates) or liver.

The high presence of G2-GATA4 cell lineage in the endothelium of the adult heart, lungs and kidneys was confirmed by confocal microscopy (Figure 2B–D).

### 3.2. The G2-GATA4 Cell Lineage Contribution to the Cardiac Vasculature Starts During the Earliest Stages of Coronary Development and it is Related with Endocardium

**Figure 3:**
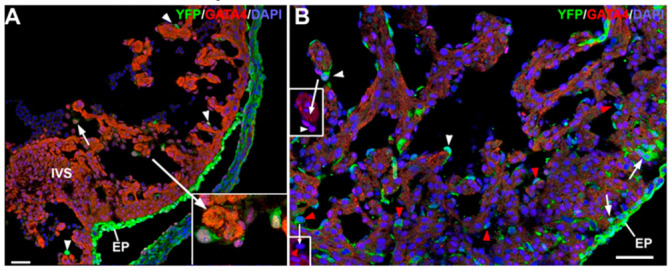
Localization of cells of G2-GATA4 lineage in the embryonic heart. (**A**) E11.5 embryo. Cells of the G2-GATA4 lineage expressing GATA4 can be seen in the circulation (arrow) and also incorporated into the ventricular endocardium (arrowheads and insert). The epicardium (EP) is also composed by cells of the G2-GATA4 lineage. (**B**) By stage E14.5, there are G2-GATA4 lineage cells in both ventricular endocardium (arrowheads) and subepicardium (arrows). White arrowheads show endothelial cells facing the ventricular lumen and expressing GATA4, while red arrowheads indicate abundant GATA4-negative endothelial cells into the intertrabecular sinusoids. Red channel (GATA4 expression) is shown in the inserts. Scale bars: 50 μm.

We have described previously the recruitment of circulating G2-GATA4 lineage cells into the ventricular endocardium, and how this process increases the fraction of the cardiac endothelium derived from this lineage. We studied when this process starts. In early embryos (E11.5) it is possible to see the first G2-GATA4 lineage cells in the endothelial lining or circulating into the ventricular cavity (Figure 3A). These cells express GATA4 and frequently show a globular shape. In later stages, these G2-GATA4 lineage cells are far more abundant, and they appear into the intertrabecular sinusoids, where their GATA4 immunoreactivity has disappeared (Figure 3B, red arrowheads). Thus, two populations of G2-GATA4 lineage cells can be distinguished in the heart by midgestation, one delaminating from the epicardium (arrows in Figure 3B) and another one related with the endocardium (arrowheads in Figure 3B).

### 3.3. The G2-GATA4 Cell Lineage Contribution to the Embryonic Endothelium of the Kidney Derives from the Lateral Mesoderm

**Figure 4:**
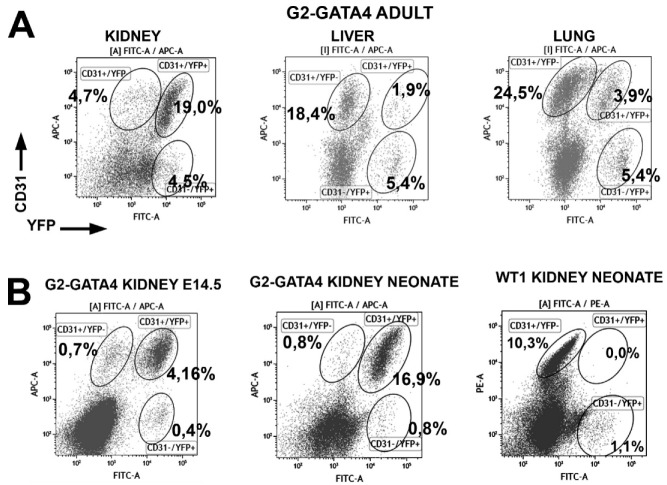
(**A**) Representative cytograms of G2-GATA4 lineage cells in kidney, liver and lungs of adults. (**B**) Representative cytograms of G2-GATA4 and WT1 lineage cells in kidneys of embryos and neonates. G2-GATA4 endothelial cells were predominant in kidneys of embryos, neonates and adults. However, there were no WT1-lineage endothelial cells in neonates. Percentages refer to total viable cells.

As described above, 92% of the kidney endothelium in neonates and 86% in adults derives from the G2-GATA4 lineage. We checked this percentage in kidneys of E14.5 embryos and we found that 80.0% ± 2.1% (*n* = 4) of the endothelium in this stage is also derived from the G2-GATA4 lineage (Figure 4). Thus, differently to that observed in the heart, the G2-GATA4 lineage accounts for the vascularization of the kidney from the earliest stages of their development, and the recruitment of circulating progenitors does not seem significant, as discussed below. Instead, angiogenesis from the lateral mesoderm adjacent to the metanephric primordium can account for the high frequency of G2-GATA4 progenitors. Figure S1 shows the ureteric buds in the metanephric primordium of an E12.5 embryo, revealing the abundance of endothelial cells from the G2-GATA4 in the adjacent lateral mesoderm.

### 3.4. Early Hematopoietic Progenitors Contribute to the Coronary Endothelium

**Figure 5:**
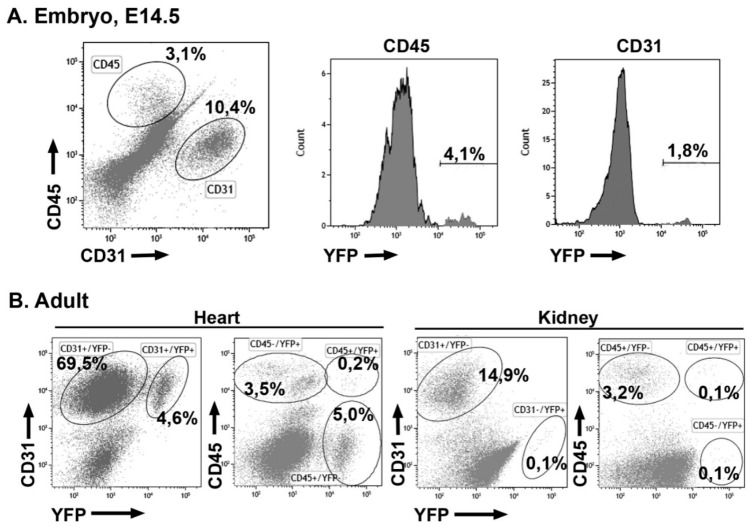
Representative cytograms of cardiac cells from the HSC-SCL^CreERT^ lineage (hematopoietic) induced at the stage E7.5. (**A**) E14.5 embryo. Some cardiac endothelial cells were YFP+, suggesting a hematopoietic origin. (**B**) Cardiac and kidney cells obtained from an adult mouse induced at the stage E7.5. Almost all the YFP+ cells in the heart were endothelial and they account for a fraction of all the cardiac endothelium. However, the kidney lacked YFP+ endothelial cells. Percentages refer to total cells.

Labeling of the hematopoietic lineage at the stage E7.5 (HSC-SCL^CreERT^; R26R^EYFP^ model) provoked that a fraction of the cardiac endothelium and cardiac hematopoietic cells became YFP+ by the stage E14.5 (Figure 5A). Namely, 2.6% ± 0.4% of the CD31+ and 6.6% ± 1.5% of the CD45+ cardiac cells were labeled (*n* = 8). In an adult mouse, 4 weeks old, also induced by the stage E7.5, we found 6.2% of the endothelial cells and 5.4% of the hematopoietic cells expressing the YFP reporter. However, in this mouse, we could not find any YFP+ endothelial cell in the kidney (Figure 5B). Thus, early hematopoietic progenitors can give rise to endothelial cells in the heart, but not in the kidneys.

### 3.5. Embryonic G2-GATA4 Lineage Circulating Progenitors can be Recruited into the Endothelium in Postnatal Stages

**Figure 6:**
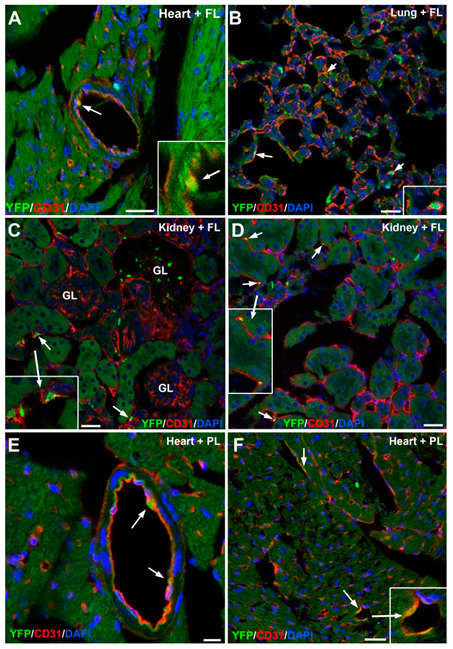
Representative images of adult organs from mice injected at the newborn stage with fetal liver (FL) and placental (PL) cells from E11.5 G2-GATA4^Cre^; EYFP embryos (panels **A**–**D** and **E**,**F**, respectively). In the heart (**A**,**E**,**F**), lung (**B**) and kidney (**C**,**D**), endothelial cells were recruited into the endothelium four months after transplantation (arrows). The inserts show higher magnification images to illustrate colocalization of markers. Scale bars: 25 μm. except for E (10 μm).

We next checked if putative circulating progenitors from the G2-GATA4 lineage can be recruited by the endothelium in postnatal stages. We injected liver or placental cell suspensions from E11.5 G2-GATA4Cre; EYFP embryos into the blood stream of neonatal mice. By this stage G2-GATA4 lineage hematopoietic progenitors can be found in both organs [5]. After 4 months, we found a number of endothelial cells in the heart, lung and kidneys (Figure 6), showing that the recruitment of circulating endothelial progenitors is still occurring in postnatal stages.

## 4. Discussion

Despite controversy concerning the different sources contributing to the coronary endothelium [15,16], it is surprising that an extracardiac/circulating source has not considered until recently [2,17]. On the other hand, it is also remarkable that most studies considering different endothelial sources have tracked the lineages until mid- or late gestation—neglecting the possibility of further changes in the composition of the coronary endothelium in postnatal stages. We already published results that show that two of the usual lineage tracing systems were not reliable for following the fate of the epicardial derived cells [17]. WT1 is expressed de novo in some cardiac endothelial cells increasing slightly the frequency of this lineage in the cardiac endothelium from the 4% obtained with other lineage-tracing systems until 7% in late embryos. We have found now that in neonates this percentage rose to 10% and remained stable until the adult stages.

In contrast with our observations on the WT1 lineage, the contribution of the G2-GATA4 lineage to the cardiac endothelium reported in our previous work [17] increased from the stage E14.5 on, reaching 20% in late embryos. We explain the increase as a consequence of the recruitment of circulating endothelial progenitors. Endomucin expression allowed us to distinguish between these progenitors and the epicardial-derived angioblastic cells. We have shown herein that the contribution of the G2-GATA4 lineage to the cardiac endothelium also increases postnatally, from 34.5% in neonates to more than 50% in adults. In contrast, the actual epicardial contribution to the adult, as shown by the GATA5 and cTnT lineages is very small, less than 2%. Furthermore, the early labeling of the hematopoietic lineage in the HSC-SCL^CreERT^ model [29] confirmed the incorporation of early hematopoietic cells to the cardiac endothelium. These results are surprising since they suggest that a major part of the adult coronary endothelium would derive from extracardiac/circulating progenitors recruited into the endocardium. However, we think that our observations are entirely congruent with previous reports on the participation of the ventricular endocardium in the coronary development [10,14], since the circulating progenitors apparently incorporate into this endocardium and presumably acquire their phenotypic features.

The postnatal period is characterized by rapid growth of the coronary vasculature due to the rapid increase in cardiac size and performance [30]. We have observed during this period an increase of the G2-GATA4 lineage contribution from neonates to adults. A plausible explanation of this increase is a differential proliferation. We have shown that the endothelial cells from the G2-GATA4 lineage proliferate more than the rest of the endothelium in neonates [17]. The more recent incorporation of these presumably more immature endothelial cells to the vascular walls would explain their higher proliferation compared with the resident, mostly quiescent, endothelium. However, a specific study must be required to characterize potential transcriptomic differences between the G2-GATA4 lineage endothelium and other endothelial cells. A second explanation, not conflicting with the preceding one, is the postnatal recruitment of circulating progenitors. In fact, this possibility is suggested by our results. Injection of fetal liver or placental cells in neonates show that G2-GATA4 lineage cells can incorporate into the endothelium of several organs, included the heart. However, we cannot estimate how relevant is this putative source of endothelial cells in physiological conditions and what part of the increase recorded between neonates and adults can be explained by this process. Endomucin expression was not useful in adult mice to identify putative circulating progenitors, as it was in embryos [17]. Thus, a temporal study of the presence of endothelial progenitors in the circulation, from embryos to adult mice, would be required to respond to this issue.

We extended our study on the endothelial contribution of the G2-GATA4 lineage to other viscera and we were surprised by the high proportion of these cells in the kidney endothelium (80%–92% in embryos, neonates and adults). In contrast, the contribution to the endothelium of other organs such as liver or lungs is moderate (10%–15%), much smaller than that observed in the heart. We think that the striking abundance of endothelial cells from the G2-GATA4 lineage in the kidneys is not due to the recruitment of hematopoietic progenitors. In the kidneys, a high frequency of this lineage is recorded since the earliest stages of their development, and we have not found a significant increase in this frequency from neonates to adults. Furthermore, we could not find endothelial cells derived from hematopoietic progenitors in the adult kidneys, using the HSC-SCL^CreERT^ model, although other studies have found a small population of renal endothelial cells from hematopoietic origin when these mice are induced at the stage E6.5 [31]. In fact, we have found a small number of G2-GATA4 lineage endothelial cells in the adult kidneys after injection of fetal liver cells in neonates (Figure 6C,D).

The issue of the development of the kidney vascularization is highly controversial [32]. Compelling evidence of sprouting angiogenesis has been provided [33]. These authors describe how the metanephric mesenchyme is invaded by angiogenic sprouts from peripheric endothelial plexuses. This can be significant, since we have shown that these perirenal vessels are mainly composed of G2-GATA4 endothelial cells. Anyhow, the authors do not discard that resident angioblasts can contribute to the renal vasculature, as suggested by other studies (e.g., [34]). Thus, we think that hematopoietic progenitors from the G2-GATA4 lineage contribute to a major part of the adult endothelium of the heart—and to a minor part of the lung and liver endothelium—but they do not participate significantly in the kidney vascularization.

In summary, we think that more attention should be paid to the importance of circulating endothelial progenitors in the vascularization of the organs, both pre- and postnatally. In recent years, the high heterogeneity of the endothelial cells has been highlighted, and this heterogeneity could be relevant from the clinical point of view (see [35,36] for a review). Endothelial cells could retain memory of their embryonic origin contributing in this way to their adult heterogeneity.

## Figures and Tables

**Table 1:** Contribution of the four lineages studied to the adult endothelium (CD31) and to the CD90 and CD140a (PDGFRα) populations of the heart (mean ± S.E.M.).

	Lineage	WT1			G2-GATA4			GATA5			cTnT		
		CD31	CD90	CD140a	CD31	CD90	CD140a	CD31	CD90	CD140a	CD31	CD90	CD140a
**Atrium**	%	22.7 ± 4.1 (*n* = 8)	10.1 ± 2.2 (*n* = 6)	20.5 ± 4.2 (*n* = 6)	29.9 ± 3.3 (*n* = 9)	9.1 ± 1.5 (*n* = 8)	19.9 ± 3.9 (*n* = 8)	30.6 ± 3.2 (*n* = 4)	7.7 ± 0.8 (*n* = 3)	21.8 ± 6.1 (*n* = 2)	26.4 ± 4.1 (*n* = 4)	10.1 ± 1.2 (*n* = 3)	18.6 ± 4.6 (*n* = 3)
	%YFP	7.9 ± 1.1	23.3 ± 4.3	56.0 ± 7.8	55.2 ± 2.8	52.2 ± 6.2	60.9 ± 10.9	0.67 ± 0.16	50.3 ± 6.8	38.4 ± 9.7	0.58 ± 0.11	20.4 ± 0.71	18.9 ± 8.1
**Ventricle**	%	60.7 ± 3.8 (*n* = 8)	6.1 ± 0.5 (*n* = 8)	6.7 ± 1.1 (*n* = 8)	72.7 ± 2.1 (*n* = 9)	6.5 ± 0.6 (*n* = 8)	4.4 ± 0.5 (*n* = 8)	58.3 ± 6.3 (*n* = 4)	6.6 ± 1.5 (*n* = 4)	3.6 ± 0.6 (*n* = 4)	60.6 ± 4.0 (*n* = 4)	6.8 ± 1.0 (*n* = 4)	6.9 ± 0.8 (*n* = 4)
	%YFP	9.9 ± 0.7	37.4 ± 2.7	31.4 ± 4.7	53.3 ± 3.1	57.4 ± 6.8	52.3 ± 5.9	1.6 ± 0.74	63.8 ± 2.5	38.7 ± 4.1	0.97 ± 0.092	35.1 ± 6.6	14.0 ± 2.7

**Table 2:** Contribution of the WT1 and G2-GATA4 lineages to the endothelium of heart, lung, liver and kidneys (mean ± S.E.M.).

		Ventricle	Lung	Liver	Kidney
**WT1 Neonates**	% CD31	11.3 ± 2.6 (*n* = 3)	6.2 ± 2.3 (*n* = 3)	8.1 ± 0.3 (*n* = 3)	15.6 ± 2.1 (*n* = 3)
	% CD31+/YFP+	10.4 ± 1.3	2.9 ± 0.9	1.0 ± 0.6	0.03 ± 0.03
**WT1 Adults**	% CD31	60.7 ± 3.8 (*n* = 8)	35.6 ± 0.9 (*n* = 3)	28.5 ± 1.09 (*n* = 3)	22.6 ± 3.8 (*n* = 3)
	% CD31+/YFP+	9.9 ± 0.7	4.1 ± 1.2	5.6 ± 1.5	0.46 ± 0.07
**G2-GATA4 Neonates**	% CD31	17.7 ± 1.6 (*n* = 12)	5.2 ± 0.8 (*n* = 7)	8.9 ± 2.3 (*n* = 7)	16.9 ± 2.1 (*n* = 5)
	% CD31+/YFP+	34.5 ± 2.3	10.8 ± 1.4	9.6 ± 2.1	92.7 ± 1.0
**G2-GATA4 Adults**	% CD31	72.7 ± 2.1 (*n* = 9)	37.8 ± 2.69 (*n* = 5)	34.5 ± 5.49 (*n* = 3)	17.8 ± 6.2 (*n* = 3)
	% CD31+/YFP+	53.3 ± 3.1	15.6 ± 1.8	9.9 ± 4.3	86.7 ± 4.7

## Supplementary Materials

The following are available online at https://www.mdpi.com/2073-4409/9/5/1257/s1, Figure S1: G2-GATA4^Cre^;EYFP embryo, stage E12.5. The vascularization of the metanephric primordium (MN) is beginning. The early vessels inside and surrounding the metanephros contain many G2-GATA4 lineage endothelial cells (arrows). AO: aorta, ND: nephric duct; UB: ureteric bud, HL: hindlimb. Supplemental Table S1. Antibodies used in this study.
