# Supplementary material for: Contribution of a GATA4-Expressing Hematopoietic Progenitor Lineage to the Adult Mouse Endothelium
Source: Cells. 2020 May 19;9(5):1257. doi: 10.3390/cells9051257 (PMC7290801; doi:10.3390/cells9051257)
Supplement: Supplementary file 1 [file cells-09-01257-s001.pdf]

Figure S1: G2-GATA4^{Cre};EYFP embryo, stage E12.5. The vascularisation of the metanephric primordium (MN) is beginning. The early vessels inside and surrounding the metanephros contain many G2-GATA4 lineage endothelial cells (arrows). AO: aorta, ND: nephric duct; UB: ureteric bud, HL: hindlimb.

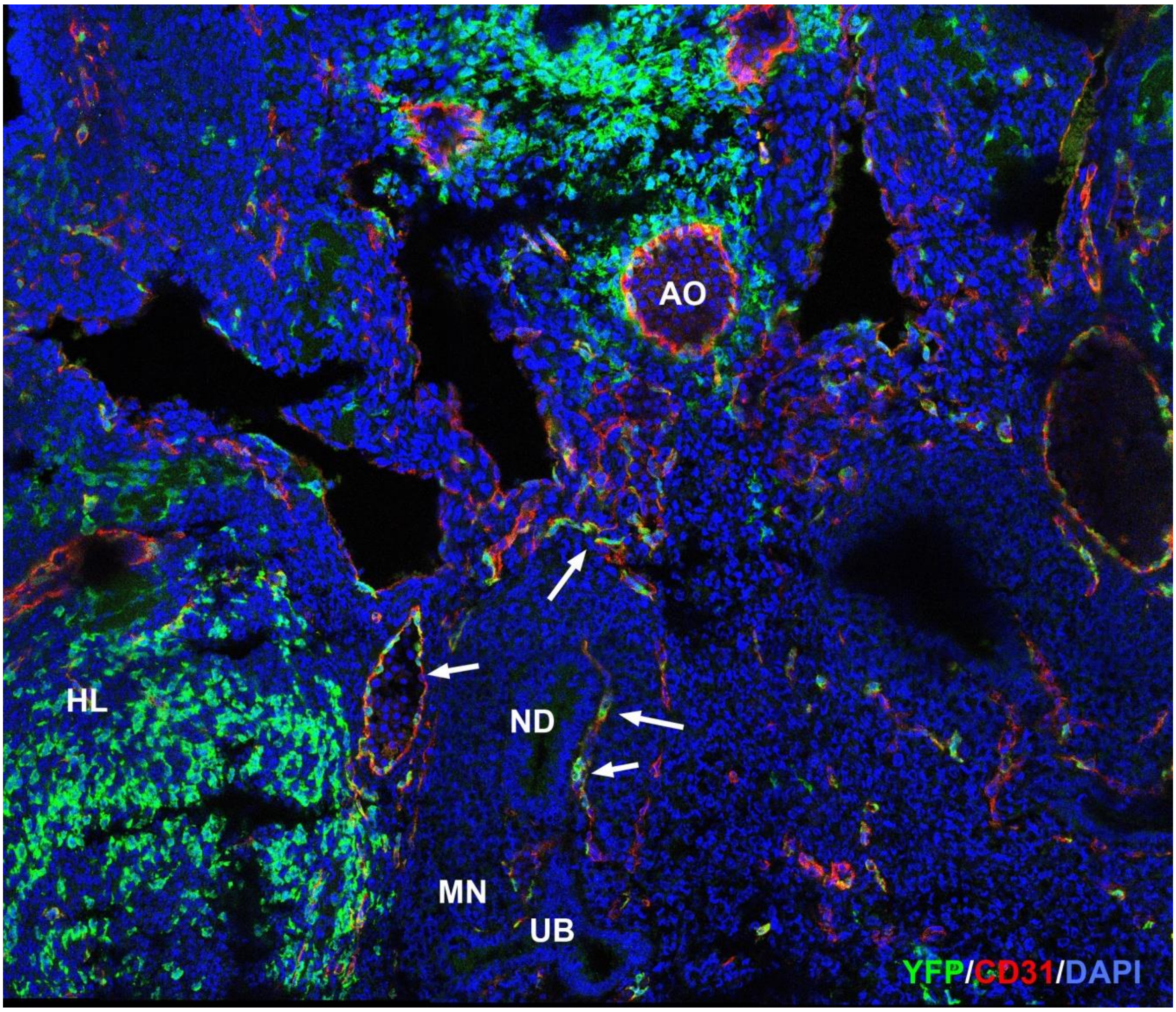

Supplemental Table 1. Antibodies used in this study

Antibody	Supplier	Clone or Ref.	Dilution
Chicken polyclonal anti-GFP	Abcam	ab 13970	1/200
Rat monoclonal anti-CD31-APC	Invitrogen	17-0311-82	1/100
Rat monoclonal anti-CD31-PE	Invitrogen	12-0311-81	1/100
Rat monoclonal anti-CD45-PE	Invitrogen	12-0451-81	1/100
Rat monoclonal anti-CD45-APC	Invitrogen	17-0451-82	1/100
Rat monoclonal anti-CD90-APC	Invitrogen	17-0902-82	1/100
Rat monoclonal anti-CD140a-APC 17-	Invitrogen	17-1401-81	1/100
Rabbit polyclonal anti-GATA4	Santa Cruz	Sc-9053	1/100
